# American Society for Enhanced Recovery (ASER) and Perioperative Quality Initiative (POQI) joint consensus statement on measurement to maintain and improve quality of enhanced recovery pathways for elective colorectal surgery

**DOI:** 10.1186/s13741-017-0062-7

**Published:** 2017-03-17

**Authors:** S. Ramani Moonesinghe, Michael P. W. Grocott, Elliott Bennett-Guerrero, Roberto Bergamaschi, Vijaya Gottumukkala, Thomas J. Hopkins, Stuart McCluskey, Tong J. Gan, Michael Monty G. Mythen, Andrew D. Shaw, Timothy E. Miller, For the Perioperative Quality Initiative (POQI) I Workgroup, Timothy E. Miller, Andrew D. Shaw, Michael G. Mythen, Tong J. Gan, Matthew D. McEvoy, Michael J. Scott, Deborah Gordon, Stuart Grant, Julie K. M. Thacker, Christopher L. Wu, Robert H. Thiele, Karthik Raghunathan, C. S. Brudney, Dileep N. Lobo, Daniel Martin, Anthony Senagore, Stefan D. Holubar, Traci Hedrick, John Kellum, Ruchir Gupta, Mark Hamilton, S. Ramani Moonesinghe, Mike P. W. Grocott, Elliott Bennett-Guerrero, Thomas J. Hopkins, Roberto Bergamaschi, Stuart McCluskey, Vijaya Gottumukkala

**Affiliations:** 1 UCLH NIHR Surgical Outcomes Research Centre and NIAA Health Services Research Centre, Royal College of Anaesthetists, London, UK; 2 Faculty of Medicine, University of Southampton, Southampton, UK; 3 Department of Anesthesiology, Stony Brook University School of Medicine, New York, USA; 4 Department of Surgery, Stony Brook University School of Medicine, New York, USA; 5 MD Anderson, Texas, USA; 6 Department of Anesthesiology, Duke University Medical Center, Durham, North Carolina USA; 7 Department of Anesthesia, University of Toronto, Toronto, ON USA; 8 Department of Anaesthesia and Perioperative Medicine, University College London, London, UK; 9 Department of Anesthesiology, Vanderbilt University Medical Center, Nashville, Tennessee USA

**Keywords:** Enhanced recovery, Enhanced recovery pathway, Outcomes, Quality, Colorectal surgery

## Abstract

**Background:**

This article sets out a framework for measurement of quality of care relevant to enhanced recovery pathways (ERPs) in elective colorectal surgery. The proposed framework is based on established measurement systems and/or theories, and provides an overview of the different approaches for improving clinical monitoring, and enhancing quality improvement or research in varied settings with different levels of available resources.

**Methods:**

Using a structure-process-outcome framework, we make recommendations for three hierarchical tiers of data collection.

**Discussion:**

Core, Quality Improvement, and Best Practice datasets are proposed. The suggested datasets incorporate patient data to describe case-mix, process measures to describe delivery of enhanced recovery and clinical outcomes. The fundamental importance of routine collection of data for the initiation, maintenance, and enhancement of enhanced recovery pathways is emphasized.

## Background

Enhanced recovery pathways (ERPs) are a structured approach to improving the quality of perioperative patient care, typically in the setting of elective major surgery. ERPs comprise a list of best practice recommendations throughout the patient pathway, which, when consistently applied, result in improved patient outcome. The unifying aim of ERPs is to minimize the physiological disturbance associated with major surgery and promotion of behaviors that will accelerate the recovery process. Successful evaluation of such quality improvement or research endeavors is critically dependent on careful selection of the measures used for evaluation. At first glance, this statement may seem disingenuous; if a treatment “works,” then surely this will be evident irrespective of the measure by which success is judged. However, ERPs may be considered a “complex intervention” where multiple stakeholders (including patients) must work together to achieve full implementation of the prescribed pathway. As with all complex interventions, therefore, it is important to evaluate compliance as well as outcome, as this may provide valuable insight into why some centers may achieve better outcomes than others (Moonesinghe 2016). This is particularly pertinent in light of evidence which suggests that there is a “dose response” effect between increasing compliance with specific ERP elements and reduced length of stay in hospital (Simpson et al. 2015). Further, the use of unvalidated and inappropriately applied outcome measures risks both over- and/or underestimation of therapeutic effect in clinical studies (Pearse et al. 2014). Finally, when the evidence supports implementation at scale, poorly selected outcome measures may lead to a loss of belief and resilience in quality improvement initiatives, which may ultimately risk undermining clinical engagement and effectiveness. The aim of this manuscript is to provide recommendations for the evaluation of ERP implementation and to describe the process by which these recommendations were reached.

## Methods

On March 4–5, 2016, the first Perioperative Quality Initiative (POQI-1) was held in Durham, North Carolina (Miller et al. 2016). A group of international experts was established, including viewpoints representing anesthesiology, surgery, and nursing. The group was divided into four workgroups focused on specific topics related to colorectal surgery within an ERP. In this workgroup, experts were asked to set out a framework for measurement of quality of care.

POQI-1 was a consensus building conference designed around a modified Delphi process in which the group alternately convened for plenary discussion sessions and then retired for small group discussion (Miller et al. 2016). Over 2 days, consensus was reached around the main issues within each topic. The group chairs and co-chairs were responsible for leading the discussions and summarizing the group topic discussions, recommendations, and suggestions for future research.

## Discussion

### Taxonomy of measures

In order to fully evaluate an ERP, quality measures should include all aspects of the program as categorized according to Donabedian’s Quality of Care Framework: i.e., structure, process, and outcome model (Donabedian 1966). Structure refers to organizational context, both “hard” features (e.g., medical equipment, pharmacy budgets, staffing schedules) and “soft” or cultural factors (i.e., organizational leadership, staffing incentives, team interactions and behaviors). Organizational context may be further divided according to levels of enquiry when conducting evaluations—macro (national or international policy), meso (institutional or regional), or micro (clinical teams). Process refers to the series of actions or steps in the delivery of care. Each of the elements of an ERP is a process that may be measured or recorded. Outcome measures should capture the result of the intervention. Candidate outcomes include patient-reported surveys (e.g., health-related quality of life, satisfaction, quality of recovery), objective and patient-centered measures (e.g., complications, mortality), measures of resource use (e.g., length of hospital or critical care stay), or economic measures (e.g., incremental cost-effectiveness ratio). Measurement of patient factors for the purposes of case-mix adjustment or comparison of baseline characteristics should also be considered.

In the context of a clinical trial of an ERP (or an individual ER element), both process and outcome should be measured. If, as should always be the case with clinical trials of efficacy or effectiveness, there are sufficient resources available to ensure that the treatment (in this case, the pathway) is delivered according to a study protocol, then an “intention-to-treat” analysis may be adopted, with important information derived from measuring protocol deviations to contextualize the outcomes.

In the case of a quality improvement (QI) initiative, process measures are likely to be of most use in driving change, but all three domains of structure, process, and outcome should be evaluated.

## What data should be collected?

Choice of data to be collected will be dependent on the setting, the aim (research vs. quality improvement), and the available resources, including staffing and technology.

## Research

The challenge of inconsistent use of outcome measures in clinical studies has driven two major international initiatives, which will be reporting in 2016–2017. COMPAC (Core Outcome Measures for Perioperative and Anaesthesia Care; http://comet-initiative.org/studies/details/632?result=true) has arisen from the COMET (Core Outcome Measures for Effectiveness Trials) initiative (www.comet-initiative.org) which aims to identify the “vital few” outcomes which should be measured in all studies, irrespective of the primary aim or outcome, so that outcomes from different studies can be compared, contrasted, and combined (e.g., within meta-analyses). A parallel endeavor, StEP (Standardized Endpoints in Perioperative care), will develop recommendations about how different outcomes should be defined and analyzed in clinical trials. Both initiatives will focus predominantly on clinical trials, rather than epidemiology or QI, but it is likely that their recommendations will be of relevance, at least in part, in other settings. Both these initiatives will cover the full range of outcome measures discussed above, and are using systematic reviews followed by Delphi consensus methods to select and define relevant measures (Myles et al. 2016).

The POQI initiative awaits the results of these initiatives as their recommendations will be evidence based, and the ambition is that researchers, peer reviewers, and journal editors embrace them. For now, we will therefore turn to the general principles of process and outcome measures which could be used in QI and research initiatives.

## Measurement for improvement

**Fig. 1:**
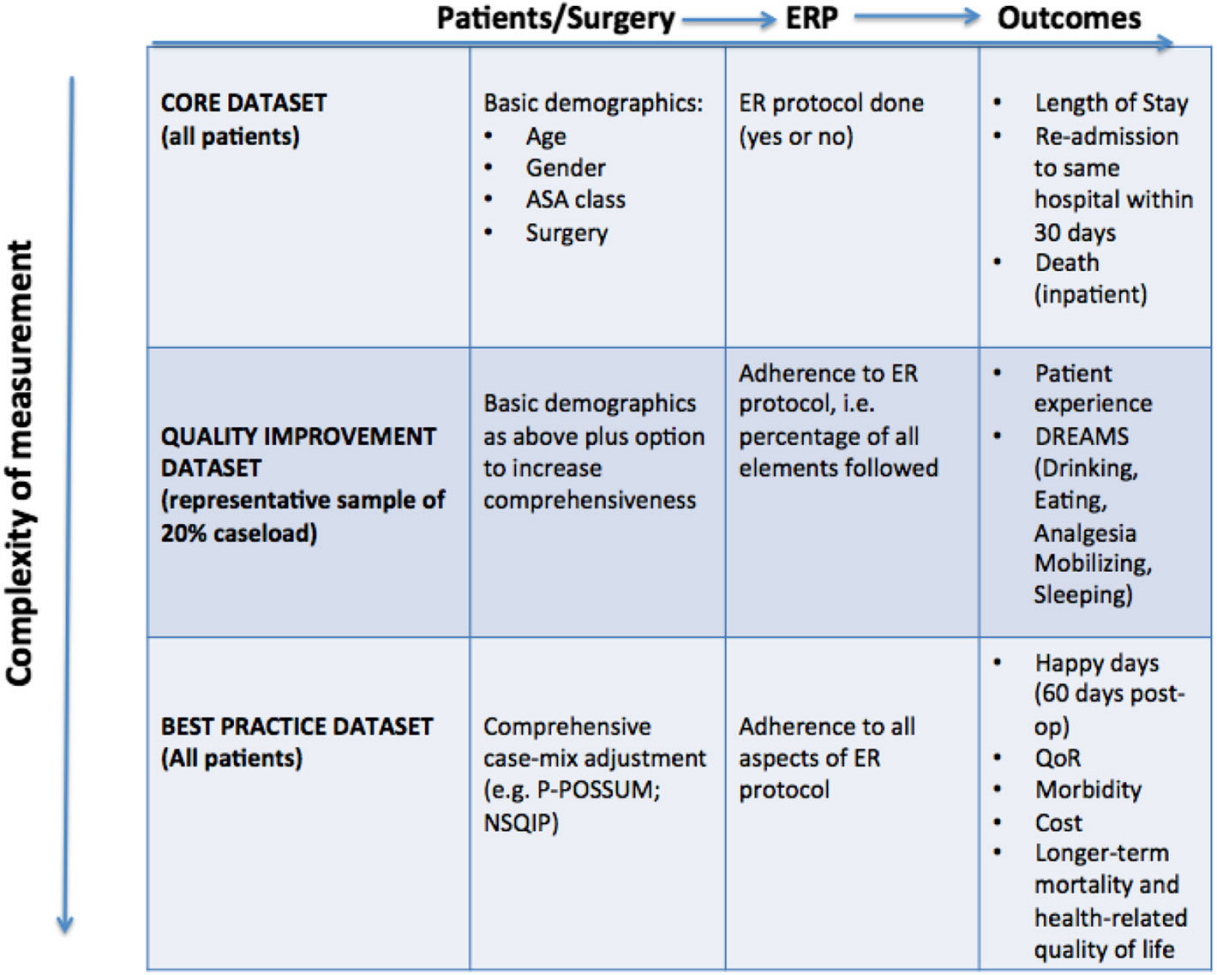
Measurement matrix for colorectal enhanced recovery pathways

Our recommendations for measurement have three tiers, which are sensitive to the resources available to local hospitals (Fig. 1). The Core dataset comprises almost entirely of routine administrative data and should be easily recordable from routine hospital data collection processes; therefore, it should be possible for all hospitals implementing colorectal ERPs to record these variables. The Quality Improvement dataset is much more comprehensive, but in keeping with the principles of measurement for improvement (Peden and Moonesinghe 2016), we recommend that this dataset is collected at regular intervals throughout the year on a sample of patients (perhaps 20% per year) so that variation in processes can be tracked. Finally, the Best Practice dataset is a comprehensive measurement and monitoring system that all hospitals may aspire to use. The collection of this dataset would likely require both technological support (e.g., electronic health record systems) and personnel support (e.g., surgical case reviewers as used in the National Surgery Quality Improvement Program (NSQIP)).

For each category of measure, we have provided a justification for the type of measure, a discussion about the issues (including evidence base) for each option, and finally our recommendations according to the three tiers described above.

## Process measures

### Justification for inclusion

ERPs are essentially a series of processes, and compliance with these processes can be used to drive quality improvement initiatives. Compliance with individual processes can be tracked over time (using run charts, e.g., percent use per month) and the nature of observed variation analyzed using statistical process control, to differentiate between common cause, or “natural” variation in the system, or special cause, i.e., unpredictable variation which requires investigation (Peden and Moonesinghe 2016). For example, if preoperative prescription of a gabapentinoid for analgesia is part of your ERP, and use of this medication drops from 90 to 30%, then there may be an explanation for the change in use that needs to be explored further.

### Measurement considerations including choice of measures

Recent work in the UK found a modest “dose response” effect between increased compliance with ERP elements and reduced length of hospital stay (Simpson et al. 2015). In keeping with the principles of industrial efficiency, high reliability (>80 or 90% of patients fully compliant) is required to effect improvement in outcomes. Thus, one could argue that external reporting of ER processes, for example, for the purposes of comparison between teams or providers, could be as succinct as simply reporting the binary indicator of whether >80% of patients received all elements of the locally approved ERP. However, by measuring individual elements, particularly those that are more challenging to implement (in UK practice, this has generally been preoperative carbohydrate loading), valuable information can be obtained for quality improvement purposes.

There may be some merit in considering ERPs as care bundles. While most ERPs are not bundles in the traditional sense (as the number of elements within an ERP usually exceeds the recommended three to five) (Resar et al. 2012), there are similarities, which are worthy of note, and raise considerations about how compliance data should be collected. There may be a tendency for clinicians to focus on the individual elements of a pathway or bundle, when contemplating whether implementation may be beneficial to their patients. However, while each element should be evidence based, it is the *package,* which should be the focus, rather than the individual interventions. The aim should be to implement *all* elements unless there is a medical contraindication, and take this as the measure of compliance (in bundle terminology—the “all or none” or AON approach) (Borgert et al. 2015).

Two further points require consideration. A recent systematic review of 50 papers relating to colorectal ERPs (Day et al. 2015) found that the level of detail regarding ER processes was generally insufficient to promote knowledge transfer. If a study either reports percentage compliance or uses the “all or none” approach, rather than reporting compliance with individual elements, it remains important that manuscripts provide detail about the pathway’s constituent measures. Finally, clinical experience and published evidence would suggest that some interventions have been straightforward to implement (e.g., in the UK, avoidance of sedative premedication) and others considerably more challenging (e.g., preoperative carbohydrate loading) (Simpson et al. 2015). This observation fits neatly within the Normalization Process Theory—that for a QI intervention to be successful, it should be coherent and encourage cognitive participation, collective action, and reflexive monitoring (May et al. 2009). When a QI intervention is evaluated as having achieved these four aims, it may be deemed to have reached its objective—that is, the process has been fully implemented and thus “normalized”. One may propose, therefore, that if an individual ER element has become so embedded that it is routine, the requirement for formal measurement may have subsided. For example, there would be little value in spending resources on measuring rates of capnography monitoring during surgical anesthesia, as this is a “normalized” process. While normalization is not necessarily irreversible, and thus some vigilance (“reflexive monitoring”) may be required to ensure that pre-implementation practice does not creep back in, omitting unnecessary process measures from data collection may renew clinical focus, enable more cost and clinically effective monitoring and QI, and, ultimately, enable a move more towards the optimal “bundled” approach to implementation.

### Recommendations

#### Core dataset

Patient-level data on entry into a colorectal enhanced recovery pathway should be recorded for the purpose of national monitoring of engagement with ER. The simplest approach would be to report the proportion of patients in an institution that are enrolled on an ERP. In institutions where patients are selected for enrolment into ERPs, as opposed to an ERP being routine practice, recording the reason for non-ERP enrolment may also be of value.

#### Quality Improvement dataset

Measuring compliance with individual elements of ERPs is important for systems monitoring. However, for quality improvement (QI) purposes, continuous measurement on a convenience sample of patients may be sufficient and can be implemented with less investment of resources than would be required for routine monitoring.

We recommend that in a sample of patients (at least 20% of annual total), compliance with individual ER elements is tracked and monitored. Participation in outcome monitoring programs such as NSQIP may facilitate this practice. Ideally, these data should be recorded on run charts to facilitate tracking compliance over time. At the beginning of implementing an ERP, compliance with all elements of the pathway in a sample of patients should be the aim. Over time, if some elements have consistent 100% compliance, local teams may consider removing these data items, as implementation has been “normalized”; at this point the focus of improvement efforts should shift to areas with poorer compliance.

#### Best Practice dataset

In hospitals where electronic health records have been introduced or there is institutional participation in outcome monitoring programs, it should be possible to record a detailed process measure dataset on all patients. Thus, best practice in process measurement, which all institutions should strive towards, is complete collection of ER compliance data, on all patients undergoing colorectal surgery, irrespective of whether they have been enrolled in an ERP or not. This level of data capture would facilitate both quality improvement and subsequent clinical research.

## Clinical outcomes

### Risk adjustment

#### Justification for inclusion

Comparison of outcomes over time or between teams or institutions would benefit from risk/case-mix adjustment or at least comparison of patient risk factors. This enables teams to understand outcomes in the context of their patients’ comorbidities and other risk factors, so enabling meaningful comparisons and avoiding the risk of biased patient selection that enhances the treatment effect.

### Measurement considerations including choice of measures

Comparison of baseline characteristics to demonstrate the similarities or differences between populations being compared could be as simple as a basic measure of functional status (e.g., the American Society of Anesthesiologists’ Physical Status Score, ASA-PSS), patient age, procedure name, and particularly if long-term outcomes are being compared, patient gender. Such data can provide baseline information without the need for regression analyses required for risk adjustment.

A systematic review of risk prediction/adjustment measures validated in heterogeneous perioperative populations evaluated the literature from 1980 to 2011 and concluded that the Portsmouth-Physiology and Operative Severity Score for the enUmeration of Mortality (P-POSSUM) and the Surgical Risk Scale were the most consistently accurate tools that have been validated in multiple studies; however, both have limitations (Moonesinghe et al. 2013). POSSUM-related tools have been evaluated in a further systematic review in colorectal cancer surgery and, again, P-POSSUM was favored (Richards et al. 2010). An advantage of POSSUM-related tools is their non-proprietary “open source” status—the models are published, enabling any healthcare system which has sufficient resources to collect and analyze their data using this model to adjust risk. However, this openness combined with a fixed model also presents a problem. It is not surprising that a risk model’s accuracy (particularly calibration) will vary over time, as changes in standards of care and the epidemiology of patient health will also change. Therefore, ideally, bespoke models would be developed which can be updated (recalibrated and discrimination re-checked) on regular basis. The NSQIP system does this; however, as the model is not published, it is not possible for other healthcare systems to use it, or validate it on their populations, unless a subscription is paid to enter the program. Nevertheless, the dataset is well known and other systems may therefore chose to collect the constituent variables.

### Recommendations

#### Core dataset

Age, gender, ASA-PS score, and surgical procedure name should be recorded on all patients, so that these basic demographic data can be compared between populations.

#### QI dataset

In a sample of patients (at least two per week) in whom compliance and outcome data are also collected, a validated composite measure of risk should be recorded. For hospitals enrolled in the NSQIP monitoring program, this should be simply the NSQIP dataset. For hospitals not enrolled in this system, we recommend the Colorectal-POSSUM model. Over time, inter-institutional data sharing would enable bespoke models for colorectal surgery to be generated for US healthcare.

#### Best Practice dataset

A comprehensive risk-adjustment measure should be included on all patients. As above, this should be the NSQIP variables for hospitals participating in that program, or the Colorectal-POSSUM elements for those outside the NSQIP system.

## Postoperative morbidity or complications

### Justification for inclusion

Short-term (inpatient) postoperative complications may have substantial impact on longer-term survival (Khuri et al. 2005; Moonesinghe et al. 2014) and quality of life (Pinto et al. 2016). They also carry substantial cost to the healthcare system (Eappen et al. 2013; Healy et al. 2016).

### Measurement considerations including choice of measures

Complications may be measured individually (e.g., pneumonia) or as composite measures (e.g., Clavien-Dindo grading (Clavien et al. 1992; Clavien and Strasberg 2009) or the Postoperative Morbidity Survey (Clavien and Strasberg 2009)). A recent systematic review of colorectal ERPs (Day et al. 2015) found that morbidity was the most commonly measured outcome (98%), followed by length of stay (94%) and mortality (90%).

Even in studies where individual organ-specific complications are recorded (e.g., any study within the NSQIP or Veterans’ Affairs (VA) systems), unless a particular organ-specific outcome is of particular interest (e.g., respiratory outcomes in studies of ventilator-associated pneumonia bundles), many studies report composites of “all complications” or “major complications” (Abedi et al. 2009; Acott et al. 2009; Al-Refaie et al. 2010; Bush et al. 2009; Hamel et al. 2005).

### Recommendations

#### Core dataset

The collection of postoperative complications data may be too onerous for smaller providers, those without access to electronic health records or with limited resources. Therefore, we recommend that outcomes are recorded using alternate means (see under “Resource use and economic outcomes”).

#### QI dataset

Process measures that may be used as a surrogate of outcome include the DREAM measures: drinking, eating, analgesia, mobilizing, and sleeping (Levy et al. 2016). Early attainment of these goals should be considered to be markers of successful ERP. We recommend that hospitals record a point prevalence estimate of DREAM on day 1 postoperatively while patients remain in hospital, in the same sample of at least two patients per week. While this has yet to be formally validated, this measure has face validity, is a pragmatic measure which requires little resource to implement, and is patient-centered.

#### Best Practice dataset

Hospitals participating in outcome monitoring programs which include measures of morbidity or complications should review results on all patients undergoing colorectal surgery, using these data (with risk adjustment) to monitor local outcomes and compare against other similar providers. Such measures may include the NSQIP complication dataset, the validated Postoperative Morbidity Survey recorded on a particular day (e.g., day 7 following surgery) (Bennett-Guerrero et al. 1999; Grocott et al. 2007), and the Clavien-Dindo scoring system (Clavien et al. 2009) which is an estimate of the severity of deviation from an uncomplicated postoperative course recorded at the time of discharge from hospital.

## Patient satisfaction, patient experience, and patient-reported outcome measures (including health-related quality of life and disability-free survival)

### Justification for inclusion

The importance of understanding outcome from the patient perspective to be able to ascertain quality and value of healthcare is acknowledged by patients, providers, and funders.

Modern therapies, particularly those administered on the critical care unit, mean that many patients who may not previously have survived a complicated postoperative course may now leave hospital, sometimes after prolonged stay (beyond 30 days); survivors are also at risk of longer-term sequelae, both physical (Khuri et al. 2005; Moonesinghe et al. 2014; Toner and Hamilton 2013) and psychological (Wade et al. 2012; Wade et al. 2013; Wade et al. 2014; Wade et al. 2015). Thus, short-term survival, while an important metric, does not provide a comprehensive assessment of outcome, particularly from the patient perspective. Therefore, a measure of quality of life or functional status should ideally be administered to help contextualize survival/mortality.

### Measurement considerations including choice of measures

Measures that are completed by patients rather than staff may include patient satisfaction, patient experience, quality of recovery, or patient-reported outcomes. Patient experience measures have been developed in different healthcare systems to meet the particular needs of those patient populations and organizational interests; they measure the patients’ views on the whole service experience, including staff behaviors, efficiency of transit through the hospital, and quality of care. Patient satisfaction measures with anesthesia care have been systematically reviewed and their development and validity formally assessed—a list of recommendations for different settings was produced (Barnett et al. 2013). Such measures may be particularly important in the very short term (e.g., within 24 h of surgery, or even before discharge from the recovery ward).

Patient-reported outcomes may be assessed in the short term (for example, the first few days or weeks following surgery) using quality of recovery scores. These measure patients’ symptoms and deviation from usual basic activities such as mobilization and sleeping. Australian researchers have developed a series of quality of recovery scores (Myles et al. 1999; Myles et al. 2000; Stark et al. 2013; Royse et al. 2010) that have been validated and systematically reviewed (Gornall et al. 2013).

Finally, patient-reported outcome measures (PROMs) evaluate health-related quality of life before and after an intervention, and the difference is taken as a measure of the success (or otherwise) of the procedure. The post-procedure measurement is typically recorded 6 months or 1 year after surgery, both generic (e.g., the EQ-5D) and condition/procedure-specific PROMs have been developed and validated. The EQ-5D is a five-domain measure that includes questions for patients about pain, mood, mobility, ability to self-care, and ability to undertake their usual activities. The EQ-5D may also be used in health economic evaluations (see later). The interpretation of health-related quality of life (HRQOL) questionnaires may be subject to some selection bias (completion rates for follow-up questionnaires vary with patient age, socio-demographic status, and ethnicity); nevertheless, they are valid composite measures of outcome and societal cost effectiveness.

The World Health Organization’s Disability Assessment Schedule v2.0 (WHODAS 2.0) has recently been validated in a heterogeneous sample of perioperative patients, of whom approximately a third had “general” surgery (Shulman et al. 2015). The WHODAS was found to be valid and acceptable to patients; it covers both physical and emotional domains in more detail than the EQ-5D. It has yet to be validated in other surgical settings.

### Recommendations

#### Core and QI datasets

A basic measure of patient experience such as the Agency for Healthcare Research and Quality CAHPS consumer satisfaction survey (Vetter et al. 2013) is recommended for all patients or at least for the sample that are having other outcomes reported (QI dataset).

#### Best Practice dataset

Administration of a validated health-related quality of life questionnaire such as the EQ-5D or SF-36 before surgery and at 1 year postoperatively is recommended. The WHO Disability Assessment Schedule v2.0 has been validated for use in the perioperative population (Shulman et al. 2015); this may provide an important alternative, enabling the measurement of “disability-free survival” at 1 year, but currently has a less comprehensive literature supporting its use.

## Resource use and economic outcomes

### Justification for inclusion

Health economic measures are an essential component of a comprehensive outcome framework if the delivery of healthcare is to be efficient in relation to the resources allocated to it. Health economics is “the science that studies human behavior as a relationship between ends and scarce means that have alternative uses” within the context of healthcare (Robbins 1932). One aim of such study is to quantify value, defined as achieving the best possible benefit per unit cost, in order to drive the efficient use of the limited financial resources supporting a healthcare system, whatever the overall system of financial flows within that system. There is an important distinction between price, a transactional concept dependent on an agreed exchange of money between a customer and a provider, and value, which is defined by the customers’ perception of benefit. A variety of definitions of value may be used to characterize the relationship between costs and benefits, but in the healthcare setting, all are limited by the difficulty of attributing a monetary value to a health benefit. Several approaches have been adopted to describe value in healthcare including cost-effectiveness analysis, cost utility analysis, and incremental cost-effectiveness ratios (ICERs). A more detailed introduction to these methods is available elsewhere in the perioperative literature (Grocott and Mythen 2015). In the US setting, the recent change from fee-for-service reimbursement to “bundled payments” has been driven by a desire to constrain healthcare expenditure and improve value.

### Measurement considerations including choice of measures

Length of stay (LOS) is one the most commonly used measures in studies of ERPs, due to face validity and ease of measurement; further, reducing length of stay (and therefore by implication complications, and as a result costs) has traditionally been one of the key perceived benefits of ERPs. However, there remain limitations in measurement of LOS: variation in how length of stay is measured may impact comparisons, LOS may be affected by factors other than quality of perioperative care (e.g., occupational therapy or social issues), and as a surrogate for a true economic analysis of costs, it performs poorly, as there is usually a decrement in non-fixed costs as a patient recovers but remains in hospital. When analyzing length of stay data for a population, the data are likely to be skewed by a few long-stay patients. Therefore, while median LOS may be the most statistically appropriate population estimate and the most representative of what most patient’s experience, mean LOS should also be reported, in order to provide a fuller picture of the total costs associated with a service.

The number of postoperative days alive and out of hospital within a limited time frame (e.g., 60 days), colloquially known as “happy days”, is a measure that combines both length of stay (important as a surrogate of complications) and survival. A patient who died in hospital on day 2 postoperatively and a patient who had a complicated postoperative course and a 75-day LOS would both have zero happy days. Such measures avoid the misleading recording of an early postoperative death as a short length of stay.

Quality of life measures are discussed in more detail above. Health economic evaluations commonly incorporate the notion of “quality-adjusted life years” (QALYs) where 1 QALY is a year in perfect health: QALY discounts survival based on quality of life measured using measures such as EQ-5D. A key consideration for health economic analyses is the time frame over which such measures are made. Benefits of surgery may be manifest for many years after the operation and the durability of QoL improvements will have a significant impact on the cost-effectiveness evaluation if a QALY-based analysis is used.

Hospital costs assess the monetary cost of ERPs and rise with increased length of stay and complicated postoperative courses. In a bundled payment environment, costs will be balanced against provider reimbursements to calculate healthcare facility profits or losses (Healy et al. 2016; Dimick et al. 2004). A variety of methods may be used to account for costs within a healthcare institution but the general trend has been towards activity-based costing (ABC), an approach which aims to attribute the true costs of any activity to that activity (including direct costs and indirect costs or overheads). Patient-level costing is specific healthcare example of ABC that has long been used in multi-payer healthcare systems (e.g., the USA) but only relatively recently adopted in “single-payer” nationally funded systems (e.g., NHS in England). ABC offers the potential for international comparisons of the cost effectiveness of healthcare at a patient or institutional level, but unfortunately, such analyses are currently challenging in the absence of internationally agreed standards for ABC.

### Recommendations

#### Core and QI datasets

Length of hospital stay is usually routinely measured, and therefore, we recommend that this is recorded for all patients undergoing colorectal surgery. Both median (and range) and mean (and standard deviation) should be reported.

#### Best Practice dataset

Hospital costs should be recorded for all patients undergoing surgery, ideally using a recognized method of ABC, to enable comparison between the patient populations who have complicated and uncomplicated postoperative stays. Postoperative days alive and out of hospital (“happy days”, with an endpoint of 60 days) should be recorded for all patients. A measure of health-related quality of life (e.g., the EQ-5D or the SF-36) should also be recorded preoperatively and at 6 months and 1 year postoperatively, to facilitate calculation of quality-adjusted life years (QALYs).

## Conclusion

This article sets out a framework for measurement of quality of care relevant to enhanced recovery pathways (ERPs) in elective colorectal surgery. We have proposed three hierarchical tiers of data collection: core, quality improvement, and best practice. Each dataset is based on a structure-process-outcome framework of quality description. The relevant data collection tier will depend on the resources available and level of engagement within a particular institution. The suggested datasets incorporate patient data to describe case-mix, process measures to describe delivery of enhanced recovery pathways and clinical outcomes. The fundamental importance of routine collection of data for the initiation, maintenance, and enhancement of enhanced recovery pathways is emphasized.

## Abbreviations

AON: All or none
ASA-PSS: American Society of Anesthesiologists’ Physical Status Score
COMET: Core Outcome Measures for Effectiveness Trials
COMPAC: Core Outcome Measures for Perioperative and Anaesthesia Care
DREAM: Drinking, eating, analgesia, mobilizing, and sleeping
ER: Enhanced recovery
ERP: Enhanced recovery pathway
HRQOL: Health-related quality of life
ICER: Incremental cost-effectiveness ratio
LOS: Length of stay
NSQIP: National Surgery Quality Improvement Program
P-POSSUM: Portsmouth-Physiology and Operative Severity Score for the enUmeration of Mortality
PROMs: Patient-reported outcome measures
QALYs: Quality-adjusted life years
QI: Quality improvement
StEP: Standardized Endpoints in Perioperative care
UK: United Kingdom
VA: Veterans’ Affairs
